# Artificial Crowns

**Published:** 1893-07

**Authors:** D. A. Allen

**Affiliations:** Toledo. O.


### Artificial Crowns.

BY D. A. ALLEN, D.D.S., TOLEDO, O.

Abstract of a paper read before the Northern Ohio Dental Society, Akron, May, 1893.

During the last few years increasing attention has been given to this branch. This has been due to several causes. Chief among these are—1st. The ever-growing desire, upon both patient and operator, to preserve the natural organs as long as possible; 2nd. The increased facilities at the disposal of the operator; 3rd. The many advantages to be gained by the combination of porcelain and the noble metals.

There are several methods of using all porcelain, porcelain and metal, and all metal crowns. Such as the old Stockton, the more modern wood pivot, Bonwill, Logan, Parmly Brown, Foster, Weston, How, Bing, Mack, Kirk, Meriam, Richmond, Dennett, Litch, etc., etc. All being more or less useful, and each having warm advocates.

From this list, together with the various combinations of the principles of two or more of them, we see there is a large scope for the choice of the dentist for the particular case in hand; and he who uses most judgment in the choice, and most skill in applying it, will succeed best.

We believe that no one should tie himself to any one method and exclude all others, as there are cases where one method will answer better than any other, others where another has peculiar adaptability; still others where the combination of the principles of two or more are needed to produce the best results.

The utmost care and judgment therefore should be exercised to do the best possible in each case.

Upon the presentation of a case, difficulties present themselves which must be met and overcome. Among these are the condition of the stump to be crowned ; the amount of destruction it has undergone ; and the condition of the surrounding tissues.

After having cared for the health of the root and surrounding tissues, and properly shielded it from further injury, we turn our attention to the preparation of the stump for the reception of the substitute of our selection.

Here arise other difficulties, which must be contended with. They may be enumerated as extent of breaking down of the remaining crown and root; the age, sex and condition of patient ; occlusion, probable strain it will be subjected to, accessibility for manipulation, etc., which must aid us in the choice of the method which promises the most permanent results, with the least destruction of tooth substances, and most nearly restores the last part to its original usefulness and appearance.

The combination of two or more methods often being necessary to the accomplishment of the end in view.

Having made choice of the method to be pursued, thoroughness should mark every step of the procedure, as the durability and usefulness depends upon the exactness with which all the details are carried out.

Our aim should always be to save as much as possible of the remaining structure, and to do as little injury to the surrounding tissues ; and to reproduce as near as possible, the original characteristics of the member operated upon, always remembering that we can not improve on old dame Nature.

Having had considerable experience in this branch of our calling, and having had quite an extended observation of the methods and manner of preparation of roots for crowns by others than myself, I deem it proper to mention a few of the faults most commonly met with in the preparation of stumps for artificial substitutes and the adjustment of the crown to the same.

The first and most common and serious error I have to contend with is to impress upon these men the importance of properly shaping the roots. These are almost invariably left nearly if not quite the original size and shape, which makes it impossible to give a crown its proper contour, and defeats the object of retaining the interdental space, and makes the crown too large at the cervical margin ; larger by twice the thickness of the gold used.

This fault also prevents the crown from fitting at its lower edge, where it is most essential it should fit as perfectly as it is possible to make it.

The root should be trimmed straight on its sides, or slightly slanting, so that the exposed end will represent a truncated cone, leaving plenty of space on the proximal sides to allow for the thickness of gold and the interdental space, which should always be maintained.

Another serious fault is putting the crown too far under the gum. This not only cuts the attachment loose from the root, to the extent that it goes beyond the free margin, but sets up peridental irritation, which sooner or later loosens the root, and the case fails.

In most cases it is necessary to remove all of the enamel remaining on the root, and in cases where the adjoining teeth have moved together and partially closed the space it is a good practice to remove considerably more, unless it is practicable to move the encroaching teeth back to their original position.

The lower edge of the crown should conform with the outline of the attachment of the free margin of the gum, and should go no farther. It should also be made thin at the edge and burnished smooth, and gradually swell out as it rises from the gum, an exact copy of a typical natural crown, of the class it represents, and knuckling with its fellows and articulating fully with the opposing organs of the opposite jaw.

Another serious fault with a great many operators is the fact that they do not let crowns come into full articulation. They should generally be allowed to articulate fully with the opposing fellows, or they will work out from the socket and expose the edge of the crown, and thereby loosen the root to that extent and also render it more liable to deleterious influences from contact with food and the oral secretions.

Another important matter in connection with this subject is the gold used where gold crowns are to be set, or bands are to be adjusted to roots for the mounting of porcelain crowns with metal base.

Pure gold is advocated by many expert crown workers, but this, we believe, is not the best form for the purpose.

Although pure gold, it is asserted, can be burnished up to the root with a small burnisher held in the fingers (oftener it is stretched instead), the enormous power of the jaws, as compared with that of the fingers, it can readily be seen, will soon stretch the band and thereby loosen the crown.

It is my opinion that gold finer than 22 k. should not be used and should be quite thick.

If pure gold is used, it should be reinforced with gold that is stiffer or with solder.

Never rely upon making a fit by the use of a burnisher, but make it fit before setting it upon the root. The burnisher is very delusive.

Finally, our aim should be to see, not how quick or how cheap, but how perfect and how durable we can do whatever we undertake in this most useful but shamefully abused branch of our art, thereby maintaining the high reputation that we "American dentists" have rightly gained, being acknowledged in all countries as being the first in the whole world, and putting to shame those pretenders and charlatans whose only aim is to fill their pockets without one thought of the obligation of operator to patient, in rendering the very best services possible and thereby extending the period of usefulness of an organ which serves a great purpose in the human economy.

